# Supplementary material for: Are carbon emissions trading and green financial instruments synergistic? -Comprehensive quantitative research based on content analysis
Source: PLoS One. 2024 Mar 7;19(3):e0298601. doi: 10.1371/journal.pone.0298601 (PMC10919601; doi:10.1371/journal.pone.0298601)
Supplement: S1 Data — (ZIP) [file pone.0298601.s001.zip › Data/地级市Z_F工作报告.zip!/║╙─╧/╨φ▓2╩╨/└┤╫╘╣1⁄2╓┌║┼í╛┬φ┐╦╩2╛▌═°í┐_2007.docx]

各位代表：

现在,我代表市人民政府，向大会作工作报告,请予审议,并请各位政协委员和其他列席人员提出意见。

一、过去六年工作回顾

市四届人大一次会议以来，在中共许昌市委的正确领导下，全市人民以邓小平理论和“三个代表”重要思想为指导，认真落实科学发展观，开拓进取，真抓实干，圆满完成了本届政府任期内的各项目标任务，实现了经济社会发展新跨越。

过去六年是经济实力大幅提升的六年。预计2006年全市生产总值717亿元，比2000年翻了一番，年均增长12.7%；人均生产总值15900元，比2000年翻了一番；财政一般预算收入25.5亿元，是2000年的3.3倍，年均增长22.1%;全社会固定资产投资311亿元,是2000年的4倍，年均增长22.4%；规模以上工业增加值278亿元,是2000年的3.9倍，年均增长18.8%;社会消费品零售总额169亿元,是2000年的2.1倍，年均增长12.7%；金融机构各项存款余额404.7亿元,比2000年增加236.4亿元,各项贷款余额303.4亿元,比2000年增加162.6亿元。

过去六年是发展后劲显著增强的六年。六年间相继开工建设了481个事关全局和长远发展的重点项目，累计完成投资294亿元。经济结构调整成效明显，二、三产业比重提高了6个百分点，高新技术产业增加值占工业增加值的比重达到21%。工业结构进一步优化。“一城三基地”建设初见成效，电力装备、烟草、金刚石及制品、发制品、食品等支柱产业、优势产业不断发展壮大；以许继集团为依托的国家（许昌）电力电子系统产业园落户我市，许昌经济开发区等9个工业集聚区成为新的经济增长点；黄河集团等5户企业被确定为国家级重点高新技术企业，省级以上名优产品74个，荣获“全国质量兴市先进市”。建设现代农业迈出新步伐。被列为首批国家农村信息化综合信息服务试点市；建立了国家农业科技园区，实施各类项目147个；培育市级以上农业产业化龙头企业65家，荣获“全国农业产业化经营先进市”；优质小麦、花卉苗木、中药材种植面积分别达到220万亩、59万亩、38万亩；畜牧业产值占农业总产值的比重达到41%。交通能源支撑能力显著增强。许平南、许尉高速公路建成通车，国、省道干线公路总里程达到644公里；禹州电厂一期建成运营，东城区热电厂开工建设，全市年发电能力比2000年增加了4.2倍；煤炭资源得到有效整合，利用效率明显提高。许昌学院新校区、市疾控中心等一批社会事业项目顺利实施，旅游、商贸物流、房地产等服务业健康发展。

过去六年是城乡面貌发生巨大变化的六年。修编了《许昌市城市总体规划》，理顺了市区规划管理体制。实施经营城市战略，建成了环城快速通道、市区污水管网环通、天然气利用等120项重点工程，新建、改造、整治了一批城市道路、背街小巷、游园广场和居民小区，累计完成投资101.6亿元。集中开发建设了东城区，交通、电力、通讯等基础设施日趋完善，城市框架基本形成。市区建成区面积51平方公里，全市城镇化率达到34%。“创三城”活动扎实有效，“碧水蓝天”目标基本实现，被命名为“中国优秀旅游城市”、“国家园林城市”。县（市）城区建设步伐加快，规模不断扩大，小城镇建设特色明显。农村基础设施更加完善。农业综合开发取得显著成效。“村村通”工程全面完成，新建、改建农村公路4445公里。实施农村饮水安全工程61处，解决了8.6万人饮水安全问题。推广农村清洁能源，建成沼气3.2万口。完成了农村电网改造工程，实现了城乡用电同网同价。推进“万村千乡”市场工程建设，建成标准农家店426个，农村消费水平不断提升。

过去六年是人民群众得到较多实惠的六年。预计2006年城镇居民人均可支配收入9010元，比2000年增加4599元，年均增长12.6%；农民人均纯收入4188元，比2000年增加1688元，年均增长9%。实现了农业税全免，落实了粮食直补、良种补贴、农机具补贴等惠农支农政策，政策性减轻农民负担8.4亿元。累计转移农村富余劳动力实现就业162万人次。新增城镇就业岗位18.7万个，8.3万名国有企业下岗失业人员实现了再就业，被国务院命名为“全国再就业工作先进单位”。城镇职工基本养老保险、失业保险、医疗保险、工伤保险、生育保险和城镇最低生活保障、廉租房制度等稳步推进，建立了农村贫困人口最低生活保障制度，社会保障体系逐步健全。坚持每年为群众办成一批实事，人民群众的生产生活条件得到不断改善。

过去六年是改革开放取得突破性进展的六年。全市国有集体企业产权制度改革基本完成，事业单位改革积极稳妥推进。农村综合改革迈出坚实步伐，行政管理、财税、金融、投资、粮食流通等项改革取得重大进展。切实提升行政效能，加强执行力建设，政府管理创新取得显著成效，行政审批事项由2000年的2035项精简到257项，精简比例达87.3%，建立了市、县、乡、村四级为民服务全程代理网络。非公有制经济快速发展，占生产总值的比重由27.2%提高到68.8%。注重利用资本市场，境内外上市公司发展到5家。六年来实际利用外资1.7亿美元，实现进出口总值20.5亿美元，其中出口17.5亿美元，对外开放迈出了新的步伐。

过去六年是社会事业全面发展的六年。积极实施“科教兴市”战略，完成各类科技计划项目1569项，申请专利1850件，科技进步对经济增长的贡献率达到50%，荣获“全国科技进步先进市”。“普九”成果得到巩固提高，农村中小学实行了“以县为主”的管理体制，改造D级危房16.4万平方米，享受“两免一补”的贫困家庭学生达到41.3万人次。普通高中教育规模不断扩大，职业教育、高等教育较快发展，组建了许昌职业技术学院，许昌师专升格为本科许昌学院。新型农村合作医疗试点工作扎实推进，有效地防控了“非典”、高致病性禽流感等重大疫情，较好地开展了艾滋病、结核病等重大传染病的防治工作。实施乡镇卫生院改造项目72个，建成了市中心血站、市紧急救援中心等15个医疗卫生项目。文化事业和文化产业全面发展，新增国家重点文物保护单位6处，被命名为“中国三国文化之乡”、“中国陶瓷文化之乡”、“中国腊梅文化之乡”。人口和计划生育工作成绩显著，连续6年受到省委、省政府表彰。环境保护、国土资源管理工作力度加大，可持续发展能力明显增强。“双拥”和军民共建活动深入开展，军政军民关系更加密切，蝉联“全国双拥模范城”。民族宗教工作成绩显著，被命名为“全国民族团结进步模范集体”。审计、统计、广播电视、体育、外事、侨务、档案、史志、人防、气象等工作都取得了新的成绩。

过去六年是精神文明和民主法制建设进一步加强的六年。广泛开展了群众性精神文明创建活动和社会主义荣辱观教育，全民素质和文明程度不断提高，先后创建省级以上文明单位103个。认真执行市人大及其常委会的决议、决定，自觉接受人大的法律监督、工作监督和政协的民主监督，累计办理人大代表议案、建议和政协委员提案1776件，办复率达100%。加强同各民主党派、工商联、无党派人士和人民团体的联系，虚心听取各界人士的意见、建议。顺利完成了第五届村民委员会换届选举。高度重视，认真处理信访问题，维护了社会稳定。严打整治斗争成效明显，社会治安防控体系更加完善，人民群众的安全感进一步增强。安全生产和食品药品监管工作进一步加强，腐竹市场专项整治和打击假冒商标卷烟工作取得了阶段性成效。认真落实党风廉政建设责任制,切实纠正行业不正之风，加大案件查处力度，廉政建设不断得到加强。

各位代表，上述成绩的取得，是省委、省政府和市委正确领导的结果，是市人大、市政协监督、支持的结果，是全市人民共同努力、苦干实干的结果。在此，我代表市人民政府，向工作在全市各条战线的工人、农民、知识分子、干部、人民解放军指战员、武警官兵，向关心支持我市现代化建设的各界人士和朋友表示衷心的感谢和崇高的敬意！

回顾过去六年的工作，我们的主要体会：一是必须坚持科学发展创新发展模式。始终把发展作为第一要务，把科学发展作为根本原则，紧密结合许昌实际，注重转变经济增长方式，注重优先发展工业、优先发展市区，注重项目建设和招商引资，走符合许昌市情的科学发展道路。二是必须坚持以人为本解决民生问题。把人民群众的利益放在首位，完善为人民群众办实事办好事的长效机制，高度关注并切实解决好热点、难点问题，让人民群众共享改革、发展成果。三是必须坚持改革创新增强发展活力。把发展的着力点放在深化改革上，坚定不移地推进体制创新、机制创新和管理创新，以改革的办法破除体制障碍，以创新的思路破解发展难题，不断增强发展的活力和动力。四是必须坚持优化环境提供发展保障。始终把强化服务、提升效能、优化环境摆在突出位置，着力打造加快发展的平台，不断增强软实力。这些体会，是本届政府在六年工作实践中的探索和总结，是对以往工作经验的继承和发展，也是今后政府工作继续遵循和把握的基本原则。

在肯定成绩的同时，我们还清醒地看到，全市经济社会发展中还存在一些突出矛盾和问题，主要表现在：产业结构不尽合理，服务业发展相对滞后；市区经济发展相对缓慢，辐射带动能力不强；出口产品结构单一，利用外资规模较小；安全生产、资源约束问题比较突出；发展环境有待进一步优化，行政效能需要进一步提升；特别是按照科学发展观的要求，切实转变经济增长方式，促进经济社会协调发展的任务还十分艰巨。对此，我们必须高度重视，采取得力措施，切实加以解决。

二、今后五年的总体要求和目标任务

今后五年，是许昌经济社会各项事业实现新发展的关键时期，面临着难得的历史机遇。国际国内产业转移加快，为我们拓展资源配置空间、承接产业转移提供了更为有利的条件；中央实施促进中部地区崛起和建设社会主义新农村重大战略，省委、省政府实施“两大跨越”、推进中原城市群建设重大部署，为我们提供了新的发展契机；许昌正处于工业化、城镇化和农业现代化快速发展时期，具备良好的发展基础。我们必须认清新形势，抢抓新机遇，发挥特色优势，依托比较优势，培育后发优势，实现科学发展、和谐发展、加快发展。

按照市五次党代会提出的指导思想和目标任务，今后五年政府工作的总体要求是：以邓小平理论和“三个代表”重要思想为指导，全面落实科学发展观，加快构建社会主义和谐社会，紧紧围绕富民强市目标，多做打基础、利长远、促发展、惠民众的事情，着力在调整经济结构和转变增长方式中实现科学发展，着力在加强资源节约和环境保护中实现持续发展，着力在推进改革开放和自主创新中实现加快发展，着力在促进社会进步和解决民生问题中实现和谐发展。

今后五年的奋斗目标是：实现国民经济又好又快发展和社会全面进步，使经济增长高于全省平均水平，城乡居民收入增长高于全省平均水平，努力实现综合实力新跨越、人民生活新提高、城乡面貌新变化、和谐社会新发展，把许昌建设成为中原强市。

——在经济发展方面：到2011年，全市生产总值突破1400亿元，财政一般预算收入突破50亿元，分别在2006年基础上翻一番；二、三产业比重达到90%。

——在城乡发展方面：2011年城镇化率达到42%以上。集中发展中心城市，市区建成区面积达到80平方公里，人口超过80万人。加快城乡一体化推进区建设。大力发展县域经济。完善县（市）城区功能，发展一批特色镇。

——在可持续发展方面：人口自然增长率控制在6.5‰以内；科技进步对经济增长的贡献率达到54%；万元生产总值能耗在2006年的基础上降低20%；全市绿化覆盖率达到40%以上，市区建成区绿化覆盖率达到42%以上；出境断面水质达标率100%，市区空气质量优良天数超过320天；市区生活垃圾无害化处理率达到100%。

——在人民生活方面：城镇居民人均可支配收入和农民人均纯收入年均分别增长9%、6%；城镇新增就业人员20万人，城镇登记失业率控制在4%以内；城镇人口基本养老覆盖人数、城镇职工参加医疗保险人数和失业保险参保人数均达到30万人以上；农村新型合作医疗覆盖率达到100%；形成以最低生活保障为基础、社会捐助为补充、专项救助相配套覆盖城乡的新型社会救助体系。

实现上述目标，事关全市人民福祉，事关许昌现代化建设全局，事关许昌在全省和中原城市群中的地位。各级政府要始终牢记使命，不负众望，以务实的作风、开拓的精神、过硬的措施，建设产业许昌、宜居许昌、活力许昌、和谐许昌、生态许昌和文明许昌，实现全市经济社会又好又快、跨越发展。

三、2007年政府主要工作

根据今后五年的目标和任务，2007年的主要预期目标是：生产总值增长12%；工业增加值增长15%；全社会固定资产投资增长20%，其中城镇以上固定资产投资增长25%；全社会消费品零售总额增长13%；进出口总额增长17%，其中出口总额增长18%；地方财政一般预算收入增长18%；农民人均纯收入增长6%，城镇居民人均可支配收入增长12%；人口自然增长率控制在5‰以内；城镇登记失业率控制在4%以内；万元生产总值能耗降低4%。

围绕上述目标，今年重点做好以下七个方面的工作：

（一）坚持产业立市，努力转变经济增长方式

突出发展二、三产业，注重提升第一产业，不断优化经济结构，转变增长方式，坚持速度、质量和效益的统一，实现又好又快发展。

着力推进新型工业化。坚持优先发展工业、优先发展市区，把大力发展工业作为富民强市的重要举措。积极推进“136”工业体系建设，着重发展装备制造、食品、纺织、能源等支柱产业和烟草、金刚石及制品、发制品等特色优势产业。集中扶持40户重点企业、100户“小巨人”企业和20户重点高新技术企业发展，争取更多的企业成为省重点企业。扎实开展“名牌兴市”活动，催生一批名牌产品和知名企业，促进品牌经济发展。实施节水、节能、节地、节材和资源综合利用等20项重大工程，加快9个工业点源综合治理，大力发展循环经济，促进经济清洁发展、节约发展、可持续发展。

大力发展集群经济。按照布局集中、产业集群、土地集约、要素集聚的原则，把许昌经济开发区等9个重点工业集聚区规划好、建设好，切实提高项目入驻率，争取集聚区工业增加值占全市规模以上工业增加值的比重提高6个百分点。制定产业集群发展规划及配套政策，提高废旧金属、煤化工、陶瓷、腐竹、棉短绒等23个产业集群的整体实力。建设许昌至长葛集群经济产业带，扩大建设规模，提升综合效益，推进许昌与郑州的产业对接、空间对接，更好地发挥辐射带动作用。

加快发展服务业。大力发展现代商贸业，努力引进一批国际国内知名的商贸企业落户许昌。积极推进万里物流园、万通汽贸城、上海商贸城、农机具交易市场等重点项目建设，着力发展仓储物流业。加快鄢陵花木、禹州中药材等专业市场建设，推进“万村千乡”市场工程。加快发展文化旅游业，挖掘曹魏文化、生态文化和钧瓷文化资源，改造提升灞陵桥景区、鄢陵花木博览园，建设钧官窑遗址博物馆，培育推出“七点一线”精品旅游线路，争取开工建设曹魏文化主题公园，形成一批高品位的文化旅游景点、景区；加强旅游产品市场营销，建立文化与旅游相结合、市场化运作的发展机制。积极发展金融、信息产业，规范引导房地产业健康发展。

做强做大县域经济。加强分类指导，优化发展环境，鼓励各县（市）结合实际，发挥优势，转变增长方式，壮大支柱产业，培育特色经济，增强县域经济综合实力。支持禹州市、长葛市围绕全国百强县目标在全省20强县（市）中前移位次，许昌县、鄢陵县、襄城县围绕全省20强目标争先进位。继续坚持并不断完善全市加快发展、科学发展新闻发布会制度，健全考评机制，充分调动各地促进发展的积极性。

（二）统筹城乡发展，扎实推进社会主义新农村建设

以发展农村经济和增加农民收入为重点，坚持公共财政向农村倾斜、基础设施向农村延伸、社会保障向农村覆盖、城市文明向农村拓展，扎扎实实建设社会主义新农村。

加快发展现代农业。在稳定提高粮食、烟叶生产能力的基础上，大力推进农业结构调整，巩固扩大花卉苗木、中药材等特色种植业规模，注重提升品质、培育品牌。大力发展以农产品精深加工为主的农业产业化经营，培植壮大20户重点农业龙头企业。加快发展现代畜牧业，认真抓好畜牧生态科技示范园和优质生猪、优质家禽、畜产品加工基地建设。大力推进农业标准化建设，建立完善农产品质量监管体系。继续实施优质粮食产业工程和沃土工程，运用现代科技和先进适用技术改造传统农业，提高农业科技含量和现代化水平。加快农业信息化建设，逐步形成市、县、乡、村相互贯通的农业信息化体系。

多渠道促进农民增收。坚持“多予、少取、放活”的方针，继续实施惠农政策型、结构调整型、科技投入型、劳务输出型、龙头带动型“五型”增收。加快转移培训农村劳动力，全年培训3万人，新增转移、输出农村富余劳动力8万人。鼓励外出务工人员回乡创业，带动农民致富。提高农民非农收入和现金收入在人均纯收入中的比重。加大扶贫开发力度，加快贫困人口脱贫致富步伐。

加强农村基础设施建设。以村庄改造为载体，以饮干净水、走平坦路、建沼气池、用卫生厕为重点，大力推进农村基础设施建设。实施农村饮水安全工程，解决7万人的饮水安全问题。巩固提升“村村通”，积极推进“村互通”，新建、改造农村公路500公里。加大沼气建设力度，新增沼气4万口。全面推进农村绿化，重点抓好山区绿化、水源地绿化和道路绿化。加强农业综合开发，切实抓好农业机械化和农田水利基本建设，提高农业综合生产能力。

积极推进许昌至长葛城乡一体化推进区建设。坚持拉开框架、有序建设、突出特色、务求实效，促进各项工作扎实开展。加快以道路为重点的基础设施建设，许昌至长葛城际通道建成通车；突出生态绿化建设，集中打造生态文化品牌；加强社会事业建设，逐步建立城乡一体的产业发展、公共设施、就业服务和社会保障体系，使推进区在全市统筹城乡社会发展中先行一步，在全市社会主义新农村建设中示范带动。

（三）实施项目带动，着力增强发展后劲

以重点项目建设为着力点，突出工业项目、基础设施项目和社会事业项目，不断强化加快发展、科学发展的项目支撑。

强化工业项目建设。着力抓好100个重点工业项目建设，力争当年完成投资80亿元，尤其是抓好50个市本级重点工业项目建设，争取当年完成投资40亿元。加快建设东城区热电厂、泉店煤矿、一林纸业文化用纸、豫中纺织喷气织机、裕丰纺织四期扩建、黄河集团技改、远东传动轴异地扩建、中锦水泥、首山焦化等项目，实现竣工投产；重点抓好烟草薄片、许昌铁矿、许继电气城二期、泉店180万吨精洗煤、奔马公司低速载重汽车、鄢陵铭达锌业、宏腾纸业15万吨白卡纸、众品公司熟食制品等项目建设，努力形成施工高潮；争取开工建设禹州电厂二期工程。积极谋划一批技术含量高、发展潜力大的重大工业项目，扎实推进项目前期工作。

推进交通设施项目建设。着力抓好17个重点交通设施项目建设，完成投资30亿元。确保许昌至禹州、禹州至登封、许昌至亳州、郑州至石人山（许昌段）等4条高速公路建成通车；确保107国道市区段改扩建和禹登铁路等项目竣工通车；争取许禹铁路技改工程等开工建设；加快实施郑武铁路（许昌段）客运专线项目；积极做好许昌至郑州城际快速通道前期工作。

加强社会事业项目建设。着力抓好16个重点社会事业项目建设，实现投资2.3亿元。完成许昌学院二期、许昌二高迁建一期等工程；加快许昌职业技术学院迁建、市会展中心等项目进度；开工建设市规划展览馆、市群众艺术馆、市残疾人康复中心、市社会福利院、河南省数字电视客户服务中心、东城区中小学校、东城区幼儿园和医院等项目；积极争取南水北调中线（许昌段）工程开工建设；建立健全广播电视“村村通”公共服务体系。积极推进市体育中心、市职工活动中心等项目前期工作。

完善重点项目推进机制。实行目标责任制，把重点项目纳入全市目标管理。严格程序，完善手续，有序推进。全面落实工程质量责任制，严格执行招标投标法，实施“阳光”工程。实行市级领导联系重点项目制度、项目法人责任制、主管部门领导责任制、项目建设进度督查制和项目建设例会制，及时解决存在的问题，精心营造良好环境，强力推进重点项目建设。

（四）坚持以人为本，大力促进社会和谐

以解决群众最关心、最直接、最现实的利益问题为重点，加强社会保障，发展社会事业，完善社会管理，推进协调发展，努力建设和谐许昌。

扩大就业与社会保障覆盖面。实施积极的就业政策，突出解决好“零就业”家庭及4050困难就业群体的就业问题，新增城镇就业人员5万人。健全社会保障体系，扩大社会保障覆盖面，妥善解决关闭破产企业退休人员的基本医疗保障和困难企业参保问题。强化城市低保动态管理，完善农村低保制度。落实廉租房制度，解决城镇低收入家庭住房问题，市区符合条件住户实现全覆盖。积极发展老龄事业，推进福利事业社会化。全面落实五保供养政策，加快推进敬老院建设，农村五保集中供养率达到30%。

加快教育、文化、卫生等社会事业发展。协调发展各类教育事业，均衡发展义务教育，提高教育质量，让更多的人享有更好的教育。大力发展农村教育，扎实推进农村中小学布局调整，不断完善农村义务教育经费保障机制，免除农村义务教育阶段学生的学杂费，为农村一批困难中小学集中配置课桌凳，新维修改造农村中小学校舍5.6万平方米。稳步发展高中教育，加快发展职业教育和高等教育，积极发展民办教育。大力发展文化事业和文化产业，抓好县级图书馆、文化馆建设及文化遗产的保护与管理，加强网吧等文化市场监管。全面推行新型农村合作医疗，巩固提高长葛市、许昌县、襄城县新农合试点成果，将禹州市、鄢陵县纳入新农合试点范围，实现全覆盖。大力发展农村和社区卫生服务，全面完成乡镇卫生院改造任务。加强体育设施建设，开展全民健身运动，提高竞技体育水平。认真落实计划生育优惠政策，持续稳定低生育水平。强化土地管理，节约集约利用土地。大力支持部队和国防建设，做好民兵预备役和兵役工作，积极开展“双拥”活动，促进军政军民团结。认真做好第二次农业普查工作。切实做好对台、外事、侨务、气象、地震、史志、档案等工作。

加强社会管理。创新社会管理体制，整合社会管理资源，推动建立政府调控机制和社会协调机制互联、政府行政功能和社会自治功能互补、政府管理力量和社会调节力量互动的社会管理网络。完善110城市应急联动服务系统和应急救援联动机制，进一步提高预防、处置突发公共事件和保障公共安全的能力。推进社区建设，完善服务功能，开展社区群众性自助和互助服务，发展社区服务业。完善村（居）民自治，支持村（居）民委员会协助政府做好社会管理工作。推进政事分开，支持社会组织参与社会管理和公共服务。

（五）加快城区建设，积极推进城镇化进程

以规划为先导，统筹发展市区、县（市）城区和重点镇，进一步提升城市综合承载能力和辐射带动能力，加快城镇化进程。

加快市区建设。按照《许昌市城市总体规划》的要求，改造提升老城区，建设完善东城区，有序开发推进区。坚持建管并重，切实做到“规划一张图，审批一支笔，建设一盘棋，管理一把尺”。健全经营城市项目策划、资金筹措、债务偿还、项目监管机制，注重完善城市功能、疏通出入市口、适当拉大框架、改造背街小巷，推进城建重点项目和基础设施建设。抓好7个续建项目和16个新建项目，建安公园、许扶运河公园、魏武帝广场、帝豪游园和天宝路西段等项目建成竣工，南北贯通延安路、魏文路，综合整治万丰路，取直文峰路南段，改造京珠高速南出入市口连接线，全年完成城市基础设施建设投资7.4亿元。

扎实推进“四城同创”。坚持把群众满意作为创建工作的根本目的，以创建“国家文明城市、国家卫生城市、国家森林城市、国家环保模范城市”为载体，坚持完善“组织领导、目标责任、属地管理、资金保障、新闻发布、考核奖惩”的创建机制，提高城市管理水平。对建安大道、莲城大道、七一路实施精细化管理，对市区主要道路、标志性建筑和背街小巷实施亮化工程。抓好道路罩面、积水点改造、人行道改造、路灯安装、交通信号灯设置、公厕建设、游园绿化、运粮河改造等21项便民、利民、惠民工程。坚持环保优先的原则，加大环保执法力度，促进生态环境建设。提高森林覆盖率，打造森林景观。确保达到创建省级文明城市目标，力争达到创建国家森林城市目标，力争通过创建国家卫生城市考核。

加快县（市）城区和重点镇建设。以强化产业支撑、完善城镇功能、改善人居环境、提高管理水平为重点，加强供水、供电、供气、污水处理、园林绿化等基础设施建设，不断提高社会发展程度。开工建设5个县（市）垃圾无害化处理等工程，不断完善城市功能。加大15个重点镇建设力度，不断加快发展步伐。

（六）坚持改革创新，不断增强发展活力

加大科技创新和成果转化力度，不断深化改革，进一步推进体制机制创新，实施开放带动主战略，努力建设充满活力的创新型城市。

坚持自主创新，提升科技水平。充分发挥政府的主导作用、企业的主体作用、人才的支撑作用、信息化的带动作用，推进自主创新和科技进步。组织实施技术创新引导工程、农村科技示范工程、知识产权战略推进工程和一批重大科技项目工程。优先发展电子信息、新材料等高新技术产业，促进优势产业和传统产业改造升级。鼓励企业加大研发投入、建立研发机构，争取使一批工程技术研究中心落户许昌。通过引进、培养等形式，着力加快创新人才队伍建设，积极培育和发展科技中介服务机构，大力推进科技成果推广转化。

不断深化改革，推进机制创新。进一步深化国有企业改革，积极引进战略投资者，加快推进许继集团产权制度改革。加强对已改制企业规范运作的指导，促进企业优化产权结构、完善法人治理结构。加大企业存量资产盘活力度，引进资金、技术、人才，增强企业发展活力。注重发展和利用证券市场，鼓励、支持企业通过多种途径上市融资。加强服务、引导，促进非公有制经济快速健康发展。积极推进教育、文化、卫生、城建等事业单位的改革改制工作。进一步深化财税体制改革，强化征收管理，提高收入质量，优化收支结构，完善国库集中支付、政府采购、乡财县管、投资评审等制度，不断扩大公共财政覆盖范围。深化农村信用社改革，扶持新组建的许昌市商业银行健康持续发展。

注重招商实效，扩大对外开放。继续加大招商引资力度，创新方式，更多地使园区和企业成为招商的主体，使产业和项目成为招商的载体，使环境和服务成为招商的优势。办好中原花木交易博览会、中国（禹州）中医药交易会、中国（许昌）国际发制品展览会，走出去，请进来，搭建平台，突出特色，努力提高招商引资实效。坚持银企洽谈会制度，加强银企合作，健全长效机制，争创全国金融生态模范试点城市。转变外贸增长方式，优化出口产品结构，培育新的出口增长点。加强对外经济技术合作，鼓励有条件的企业积极参与国际市场竞争。

（七）提高文明素质，切实加强精神文明和民主法制建设

深入开展精神文明创建活动，营造文明诚信的社会环境，推进民主法制建设，提高依法治市水平，为构建和谐社会提供精神动力和法制保障。

加强精神文明建设。以社会主义荣辱观教育为重点，加强思想道德建设，倡导爱国、敬业、诚信、友善等道德规范。积极开展文明城市、文明村镇、文明社区等创建活动，继续开展文化、科技、卫生“三下乡”和科教、文体、法律、卫生“四进社区”活动，促进城乡文明、社会和谐。

加强平安许昌建设。进一步加强信访工作，正确处理和妥善化解新时期人民内部矛盾，有效防范处置突发性和群体性事件。依法解决好土地征用、城镇拆迁、企事业单位改革改制和劳动争议等涉及群众利益的问题，努力化解社会不稳定因素。严格落实安全生产责任制，时刻敲响煤矿安全生产的警钟，切实加强交通、消防等安全监管，严肃责任追究，坚决遏制重特大事故发生。严厉打击各种违法犯罪活动，强化社会治安综合治理，争创全国社会治安综合治理先进市。

加强社会诚信体系建设。深入开展食品、药品、农资、假冒商标卷烟、腐竹生产经营、关闭取缔“十五小”和“新五小”企业等专项整治活动，严厉打击制售假冒伪劣产品的违法犯罪行为，整顿和规范市场经济秩序。积极探索建立失信惩戒制度，加快个人、企业、政府信用体系建设。严惩逃废金融债务行为，优化金融生态环境。充分发挥“12312”举报投诉热线的作用，加大知识产权保护力度。

加强民主法制建设。深入推进“五五”普法和依法治理工作，努力提高全民法律素质。自觉接受人大及其常委会的法律监督、工作监督和政协的民主监督，严格执行人大及其常委会的决议、决定，支持人大代表依法行使职权，认真办理人大代表议案、建议和政协委员提案。广泛听取各民主党派、工商联和无党派人士的意见和建议，发挥工会、共青团、妇联等人民团体的作用，保障公民特别是妇女、未成年人和残疾人的合法权益。全面贯彻党的民族宗教政策，依法做好民族宗教工作。

四、加强政府自身建设

圆满完成今年以及未来五年的各项目标任务，对政府提出了新的更高的要求。各级政府必须切实加强自身建设，把科学发展、加快发展作为第一要务，把当好公仆、为民办事作为第一职责，把狠抓落实、苦干实干作为第一选择，把依法行政、廉洁从政作为第一准则，致力建设法治型、效能型、服务型政府。

健全机制，依法行政。依法行政的水平和能力是衡量政府工作的重要标准。各级政府要严格依照法定权限和程序行使权力、履行职责、接受监督，切实把各项行政行为纳入依法运转的轨道。进一步推进行政执法责任制和执法过错追究制，加强行政复议工作，规范行政执法行为，做到严格执法、公正执法、文明执法。不断完善政府决策、执行、监督机制，健全重大事项集体决策、专家咨询和公示、听证等制度。加强对公务员的教育和培训，增强按章理事、规范运作的意识，提高依法行政、依法办事的水平。

提升效能，高效施政。强化效能建设、转变工作作风是许昌加快发展、科学发展的重要保障。深入开展“效能提升年”活动，引入先进管理理念，推进机关标准化管理，切实转变机关作风。加快电子政务建设，提高工作效率。创新工作落实机制，综合运用目标管理、绩效考评、效能监察等手段，不断提高政府执行力。完善行政责任追究制，坚决制止不作为、乱作为、有令不行、有禁不止、推诿扯皮等不良行为，确保政令畅通。

强化服务，为民执政。坚持维护群众的利益高于一切，关心群众的疾苦重于一切，解决群众的困难先于一切，真心实意为群众办实事、做好事、解难事。继续推进政府职能转变，进一步深化行政审批制度改革，切实做到审批瘦身、运作阳光、服务提质。建成启用行政服务中心，行政审批事项全部进入中心办理，做到一门受理、并联审批、限时办结。进一步推进政务公开，方便群众办事和监督。扎实开展“人民满意的公务员”评选表彰活动，增强政府公务员的公仆意识。着力办好惠农补贴、改善农民基本生产生活条件、缓解上学难看病难、就业和社会保障、困难群体救助、环境治理、市区惠民工程等关系群众切身利益的“十件实事”，切实让人民群众得到更多实惠。综合利用网络、电话、信件等渠道，畅通民情通道，实现政民互动，努力做到群众有所呼、政府有所应，群众有所需、政府有所为。

标本兼治，廉洁从政。坚持标本兼治、综合治理、惩防并举、注重预防的战略方针，加强廉政建设和反腐败工作。严格执行领导干部廉洁从政的各项规定，加强对权力运行的制约和监督，推动权力规范、透明、廉洁、高效运行。注重发挥监察、审计的职能作用，不断加大预防和治理力度，建立反腐倡廉的长效机制。坚决查处违纪违法案件，坚决惩治腐败分子，坚决纠正损害群众利益的不正之风。推进公务接待制度改革，厉行节约，杜绝浪费。始终牢记“两个务必”，大力倡树清新、简约、务本、责实的良好政风。

各位代表！

回首过去，许昌取得了非凡业绩；展望未来，许昌充满着无限生机。站在新起点、实现新跨越，是时代的要求，是人民的重托，是历史赋予我们的神圣使命。让我们在中共许昌市委的领导下，坚持以邓小平理论和“三个代表”重要思想为指导，全面落实科学发展观，励精图治，开拓进取，为实现富民强市的宏伟目标，为许昌更加美好的明天而努力奋斗！
